# VALIDATE—Utilization of the Viz.ai mobile stroke care coordination platform to limit delays in LVO stroke diagnosis and endovascular treatment

**DOI:** 10.3389/fstro.2024.1381930

**Published:** 2024-06-06

**Authors:** Thomas Devlin, Lan Gao, Oleg Collins, Gregory W. Heath, Morgan Figurelle, Amanda Avila, Caitlyn Boyd, Hira Ayub, Theresa Sevilis

**Affiliations:** ^1^Department of Neurology, Catholic Health Initiatives (CHI) Memorial Neuroscience Institute, Morehouse School of Medicine, Chattanooga, TN, United States; ^2^Department of Mathematics, University of Tennessee at Chattanooga, Chattanooga, TN, United States; ^3^University of Tennessee Health Science Center Chattanooga, Department of Medicine, Chattanooga, TN, United States; ^4^TeleSpecialists, LLC, Fort Myers, FL, United States

**Keywords:** artificial intelligence, deep learning, LVO detection, care coordination, acute stroke care

## Abstract

**Background:**

Thousands of hospitals worldwide have adopted mobile artificial intelligence (AI)-based stroke care coordination platforms. Studies exploring the benefit of these platforms have been scrutinized due to small sample size, serial cohort design, and measurement of metrics with multiple determinants. In this large multi-center study, we evaluated the ability of an AI-based stroke care coordination platform to expedite contact with the interventionalist (NIR) for potential thrombectomy.

**Methods:**

Acute stroke consultations seen by TeleSpecialists, LLC physicians at 166 facilities (17 states) utilizing Viz.ai software (AI) vs. no AI software (non-AI) were extracted from the TeleCare by TeleSpecialists™ database from December 1, 2021, through March 31, 2022. The primary outcome was time from patient arrival to first contact with the interventionalist to discuss need for potential thrombectomy (Arrival-to-NIR notification).

**Results:**

A total of 14,116 cases were analyzed. Compared to the non-AI cohort, Arrival-to-NIR notification in the AI cohort was: (1) 39.5 min faster (44.13% reduction, *p* < 0.001) in the overall analysis; (2) 33.0 min faster (34.0% reduction, *p* < 0.001) in the non-thrombectomy (non-TC) facility subgroup analysis; and (3) 34.0 min faster (43.59% reduction, *p* < 0.001) in the thrombectomy capable (TC) facility subgroup analysis. IQR range comparison demonstrated a significant improvement in uniformity of stroke workflow across all AI subgroups. Significant, albeit small, confounding biases were revealed in the data. The presence of AI within the non-TC subgroup correlated with a lower acceptance rate for thrombectomy by the NIR (delta = −10.79% absolute and 23.17% relative reduction, *p* < 0.0001).

**Conclusions:**

While this study was limited by our inability to capture detailed neuroimaging timelines and patient outcomes, it suggests a potential significant benefit of AI-based stroke care coordination platforms and underscores the critical need to development robust “big data” systems to study the effects of AI, and other emerging technologies, on stroke systems of care.

## Introduction

The rapid treatment of large vessel occlusion (LVO) stroke has been validated in short- and long- treatment window clinical trials and meta-analyses as a critical determinant of mechanical thrombectomy eligibility and good patient outcomes (Sun et al., 2013; Goyal et al., 2015, 2016; Jovin et al., 2015; Saver et al., 2015; Albers et al., 2018; Mulder et al., 2018; Nogueira et al., 2018; Carrion-Penagos et al., 2022). Previous reports indicate that factors delaying mechanical thrombectomy (MT) are complex and often driven by delay in LVO-stroke diagnosis and contact with neurointerventionalists (NIR; Sun et al., 2013; McTaggart et al., 2017; Lachkhem et al., 2018; Danziger et al., 2021; Aroor et al., 2022; Carrion-Penagos et al., 2022). In hopes of minimizing such delays, artificial intelligence (AI)-based mobile stroke platforms are now being promulgated worldwide. By combining AI-based automated LVO detection, real-time mobile high-resolution neuroimage file sharing, and multi-user communication capabilities, these platforms have been touted to accelerate stroke workflow (Murray et al., 2020; Lotan, 2021). The most utilized of these platforms are Viz.ai (VIZ) and RapidAI. Several hospitals have reported improvement in stroke workflow after implementation of such systems (Table 1; Devlin et al., 2020; Morey et al., 2021; Elijovich et al., 2022; Hassan et al., 2022a,b; Figurelle et al., 2023). Conclusions based on these past reports must be approached cautiously for three reasons. First, these studies have been primarily smaller single center studies raising concern for reproducibility. Secondly, the metrics analyzed in past studies have been complex and heavily influenced by a multitude of variables not directly under the control of the AI platform (e.g., spoke hospital door-in door-out times, time to groin puncture, length of stay, and patient outcome). Lastly, most studies have been serial cohort studies in which metrics were assessed before and after AI-based stroke platform implementation (often with years between cohorts) during which time numerous other changes in stroke workflow likely transpired. A large-scale multicenter parallel cohort study focused on stroke workflow is now warranted to assess the potential benefits of a mobile AI-based care coordination platform, better understand potential biases in the data, and assist in the design of future “big data” studies.

**Table 1:** Utilization of an AI-based system to improve acute stroke workflow summary of previous studies.^*^

	**Hospital**	**Authors**	**Title**	**Study design**	**Study period**	**Sample size**	**Main results**	**References**
1	Mount Sinai, New York, NY.	Morey J, Zhang X, Yaeger K, et al.	Real-World Experience with Artificial Intelligence-Based Triage in Transferred Large Vessel Occlusion Stroke Patients.	Restrospectie analysis of a single center hub and spoke model comparing metrics before and after VIZ implementation.	July 2018 to March 2020.	Stroke workflow was analyzed for 55 patients divided between pre-VIZ and post VIZ cohorts.	The median initial door-to-neurointerventional notification time interval was statistically faster (25.0 min vs. 40.0 min; *p* = 0.01) with less variation (*p* < 0.05) following VIZ implementation. The median initial door-to-puncture time interval was 25 min shorter in the post-VIZ cohort, although this did not reach statistical significance (*p* = 0.15).	Cerebrovasc Dis 2021;50(4):450-455.
2	University of California, San Diego., CA.	Figurelle M, Meyer D, Perrinez E, et al.	Viz.ai Implementation of Stroke Augmented Intelligence and Communication Platform to Improve Indicators and Outcomes for a Comprehensive Stroke Center and Network.	Retrospective single center Hub—Spoke model comparing metrics including door to groin puncture (DTG) before and after VIZ implementation. Assessed effect of day vs. night shift arrival.	June 2020 to June 2021	Stroke workflow was analyzed for 82 patients divided between pre-VIZ and post-VIZ cohorts.	Post-VIZ implementation: (1) faster door to groin times for patients presenting to the Spoke and Hub (HUB-DTG-24 h: 32% reduction, 127 min vs. 86 min (delta = 41); *p* = 0.006; SPOKE-DTG-24 h: 33% reduction, 42 vs. 28 min; (delta = 14), *p* = 0.036).	AJNR Am J Neuroradiol 2023;44(1):47-53.
3	Erlanger Health System—Univ. of Tennessee, Chattanooga, TN.	Devlin T, Shah R, Patterson J, et al.	(DISTINCTION): Utilization of Applied Artificial intelligence to Facilitate LVO Detection and Synchronizing Workflow to Improve Time to Treatment in High-Volume Hub and Stroke Networks.	Retrospective single center Hub—Spoke model comparing metrics before and after VIZ implementation.	Nov, 2017—Jul, 2019	Stroke workflow was analyzed for 15 patients divided between pre-VIZ and post VIZ cohorts.	Post-Viz LVO implementation, significant improvement in Spoke door-in to hub groin puncture (mean = 218 vs. 141, *p* = 0.02); and in Spoke CT to groin puncture time (mean = 200 vs. 132, *p* = 0.04).	World Stroke Organization, 2020, Abstract #3069—AS38.
4	Semmes-Nurphey Clinic, University of Tennessee, Memphis, TN.	Elijovich L, Dornbos D, Nickele C, et al.	Automated Emergent Large Vessel Occlusion Detection by Artificial Intelligence Improves Stroke Workflow in Hub and Spoke Stroke System of Care.	Retroscpective single center Hub—Spoke model comparing metrics before and after VIZ LVO implementation.	Dec, 2018—Dec, 2019	Stroke workflow was analyzed for 104 patients divided between pre-VIZ and post-VIZ cohorts.	Post-VIZ: Significant improvement in median time form CTA completion to NIR contact (delta = 19 min (26 min vs. 7), *p* < 0.001) and Spoke door in to arterial puncture for patients transferred from Spoke to Hub for EVT (delta 44 min, 185 vs. 141, *p* = 0.027).	J Neurointerv Surg. 2022 Jul;14(7):704-708. https://doi.org/10.1136/neurintsurg-2021-017714
5	Valley Baptist Medical Center, Harlingen, Texas	Hassan A, Ringheanu V, Tekle W.	The Implementation of Artificial Intelligence Significantly Reduces Door-in-Door-out Times in a Primary Care Center Prior to Transfer.	Retrospective single center Hub—Spoke model comparing metrics before and after VIZ implementation.	Feb 2017 to June 2020	Stroke workflow was analyzed for 63 patients divided between pre-VIZ and post-VIZ cohorts.	Post-VIZ median CTA time at PSC to door-in at CSC was significantly reduced by an average of 22.5 min (132.5 min vs. 110 min; *p* = 0.0470).	Interv. Neuroradiol.-−2022 Aug 25;15910199221122848. https://doi.org/10.1177/15910199221122848
6	Valley Baptist Medical Center, Harlingen, Texas	Hassan A, Ringheanu V, Preston L, et al.	Artificial Intelligence–Parallel Stroke Workflow Tool Improves Reperfusion Rates and Door-In to Puncture Interval	Retrospective single center Hub—Spoke model comparing metrics before and after VIZ implementation.	Nov 2016—May 2020	Stroke workflow was analyzed for 188 patients divided between pre-VIZ and post-VIZ cohorts.	Post-VIZ, mean door-in to puncture time at the Hub improved (delta = 86.7 min; 206.6 vs. 119.9 min; *p* < 0.001) with significant improvement in rate of reperfusion TICI 2b-3 (*p* = 0.036).	Stroke Vasc Interv Neurol. 2022;2:e000224. https://doi.org/10.1161/SVIN.121.000224

^*^The studies summarized here all investigated facilities utilizing Viz.ai (VIZ).

## Methods

**Figure 1:**
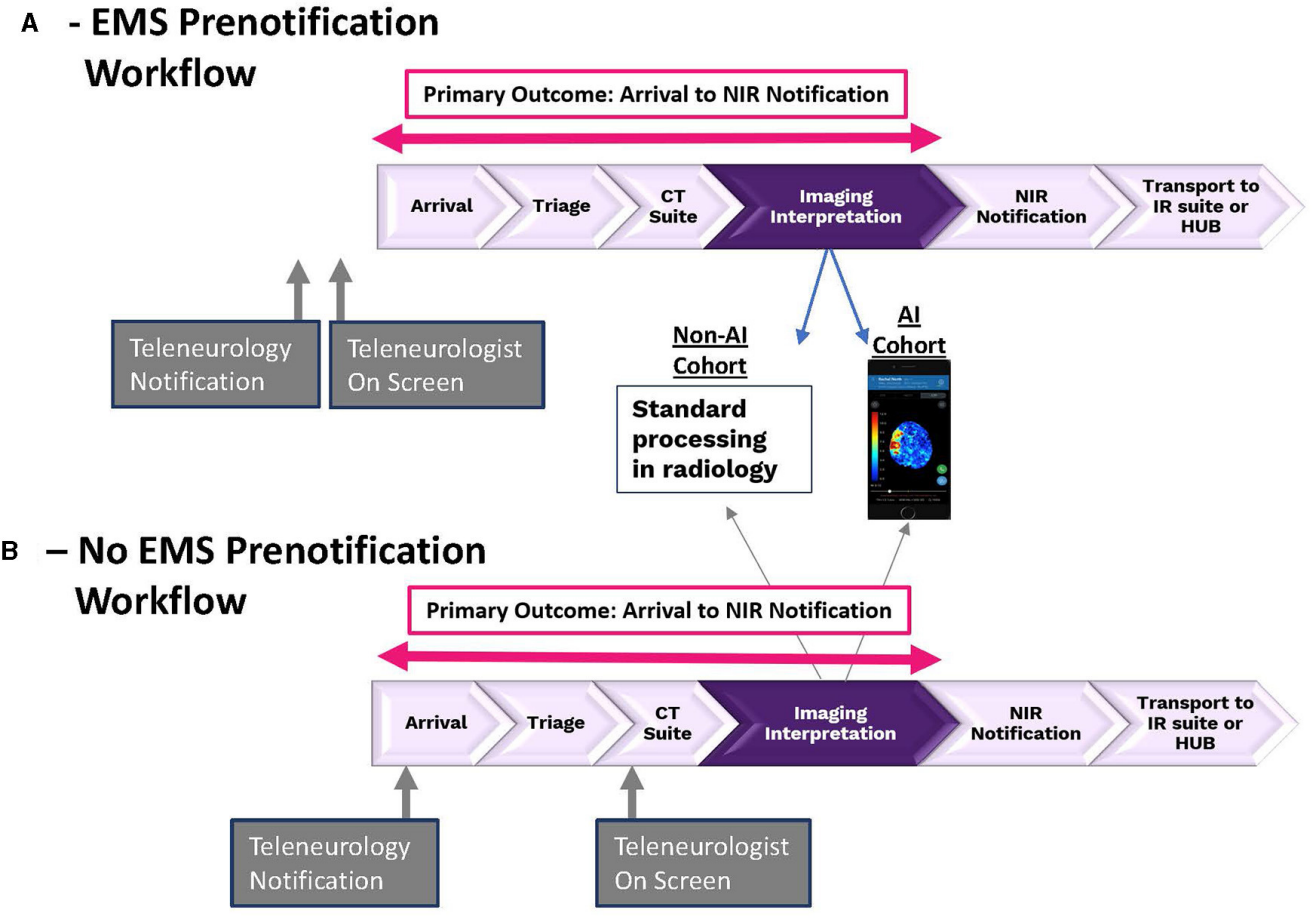
The two different stroke patient work flows within both AI and non-AI facilities. **(A)** Depicts workflow for patients in which EMS prenotification did occur and **(B)** depicts workflow for patients in which EMS prenotification did not occur.

Acute stroke consultations seen by TeleSpecialists, LLC physicians in the emergency departments of 166 facilities (17 states) that utilized the Viz.ai platform (AI) and non-AI facilities (non-AI) were extracted from the TeleCare by TeleSpecialists™ database from December 1, 2021, through March 31, 2022. Facilities using another non-VIZ AI-based platform or with protocols in which the teleneurologist did not initiate direct contact with the NIR were excluded. The same group of teleneurologists, all working remotely, evaluated patients at AI and non-AI facilities. Per the teleneurology company protocol, a 100% data entry into the web-based telemedicine database is required by all teleneurologists, including first confirmed contact time with the NIR either by phone or confirmed messaging in app, and all data must be entered before closing a case and all cases must be closed by end of shift. The two standardized workflows within both the AI and non-AI facilities are detailed in Figure 1. Figure 1A depicts the standard workflow at AI and non-AI facilities in which the stroke code involved EMS prenotification (pre-alert) and Figure 1B depicts the workflow in which no EMS prenotification occurred. The workflow at all facilities involved a series of highly standardized steps, the order of which being dependent on whether an EMS pre-alert occurred. These steps involved patient arrival and triage team evaluation in the emergency department (ED), teleneurology stroke alert activation (SAA) by the ED, immediate web-based text activation of the teleneurologist, emergent video camera login by the teleneurologist, emergent neuro imaging acquisition in radiology and viewed by the teleneurologist, and first call to the NIR to discuss possible MT in the event of suspicion for LVO on the part of the teleneurologist. In the case of EMS prenotification to the hospital, early teleneurology stroke alert activation often occurred such that the teleneurologist would arrive on camera prior to patient ED arrival. In the case of no prenotification, EMS arrival patients would be assessed by ED facility team just prior to teleneurology stroke alert activation. A similar workflow pertained to walk in patients presenting to triage. In all cases, the focus of the teleneurologist was to facilitate administration of IV thrombolysis and ensure rapid execution of advanced neuroimaging (CTA+CTP) if indicated. The primary difference in workflow between the AI and non-AI cohorts occurred with the teleneurologist and NIR viewing of the CT/CTA/CTP (see Figure 1). In the non-AI cohort, images were manually reconfigured by the CT technician and pushed to their hospital-based image reviewing platform (typically PACS) for review by the teleneurologist. In the AI cohort, neuroimaging was automatically pushed to the AI cloud-based server and made available for viewing on the teleneurologist's phone-based mobile AI platform typically within 4 min of image acquisition. By protocol, the teleneurologist reviewed all neuroimaging emergently and had the option to review imaging with a radiologist prior to emergent contact with the NIR to discuss possible MT in patients where LVO was suspected.

Our primary outcome compared the AI vs. non-AI cohorts' median value and data dispersion (IQR) for patient arrival time to teleneurologist first contact with NIR to discuss potential MT (Arrival-to-NIR notification). “NIR notification” was defined as either: (1) first direct phone contact between the teleneurologist and the NIR; or (2) the time of confirmed messaging response by the NIR and discussion on mobile AI platform. All first contract NIR notification times were entered into the telemedicine web-based database directly by the teleneurologist. We hypothesized that the metric of Arrival-to-NIR notification was less likely to be confounded by the myriad of other non-AI-platform related variables, such as referral hospital door in-door out times, thrombectomy start time or duration, length of stay, or neurological outcome. The time of first contact with NIR is typically not collected at most centers, however, an accurate timestamp was mandated by our teleneurologists per company quality protocol. Furthermore, while the retrospective analysis of this large dataset allowed us to compare certain metrics across a vast number of hospitals, the limitations of the database precluded our ability to analyze other metrics of potential interest including CT/CTA/CTP start times, thrombectomy start time, length of stay or patient outcome. We performed two prespecified subgroup analyses to control for potential influence of other variables on the results. We subgroup analyzed metrics for thrombectomy-capable vs. non-thrombectomy-capable facilities and for EMS pre-alerted vs. no EMS pre-alert cohorts.

Descriptive data analysis and inferential tests were conducted in this study. Continuous variables were summarized using means, medians, and Interquartile ranges [IQR], while categorical variables were presented as frequencies and percentages. The normality of the data was assessed using the Shapiro test. To compare continuous variables between groups, the student's *t*-test was used for normally distributed data, and the Mann–Whitney test was used for non-normally distributed data. Pearson's Chi-square test was employed for comparisons involving categorical variables. Data analytics were performed on multiple cohorts of data. Statistical significance was determined at a *p*-value of < 0.05, and all statistical analyses were conducted using the statistical package R 4.0.2. Trial design oversight and complete statistical analysis were performed according to rigorous scientific standards and best practices by faculty from the University of Tennessee Chattanooga (LG) and the University of Tennessee Health Science Center (GH) funded independently by the NeuroScience Innovation Foundation.

## Results

Data were extracted retrospectively from a total of 14,116 stroke alerts across 17 states including 8,557 alerts performed at 76 different AI facilities and 5,559 alerts performed at 90 different non-AI facilities.

### Overall analysis—AI vs. non-AI cohorts

Table 2A shows a detailed comparison between AI and non-AI patient populations. The primary finding of this study was a 39.5 min (44.13%) faster time from patient Arrival-to-NIR notification at AI [AI: 50.0 min (40.00, 82.00) vs. non-AI: 89.50 min (59.25, 122.00), *p* < 0.001]. Importantly, a statistically significant reduction was seen in the IQR in the Arrival-to-NIR in AI vs. non-AI (*p* = 0.0116; Table 2, footnote line 3). When assessing for potential biases in the Arrival-to-NIR notification times for AI vs. non-AI, a significantly faster time (2.24 min) was observed at AI facilities for patient arrival to teleneurology stroke alert activation [“Bias1,” AI: 10.32 min (4.75, 20.94) vs. non-AI: 12.56 min (6.15, 26.28), *p* < 0.001]. A small but significantly faster time (1.00 min) was also observed at AI facilities for teleneurologist stroke alert activation to first teleneurologist video login [“Bias2,” AI: 2 min (1.00, 4.00) vs. non-AI: 3 min (2.00, 5.00), *p* < 0.001]. When Bias1 and Bias2 were totaled (3.24 min), they accounted for 8.20% of the overall 39.50 min reduction in Arrival-to-NIR notification. Table 2A shows that the percentage of patients arriving by EMS vs. private vehicle was higher in AI group (55.74 vs. 51.09%, *p* < 0.001). A higher percentage of EMS pre-alerts occurred in AI cohort (AI: 24.94 vs. non-AI: 17.93%, *p* < 0.001). Of the pre-alerts, no significant difference was seen in the median time from EMS pre-alert to facility arrival between the two cohorts. While the overall percentage of patients receiving IV thrombolytics in the two cohorts was not significantly different (AI: 6.37 vs. non-AI: 6.46%, *p* = 0.86), a 4 min faster door to needle time was observed at AI facilities [AI: 40.0 min (30.00, 52.00) vs. non-AI: 44.0 min (32.00, 57.50), *p* = 0.0184].

**Table 2:** Overall analyses: AI vs. non-AI comparison for various cohorts.

		**Total**	**Non-AI cohort**	**AI cohort**	***P*-value**	**Difference**	% change^*^
**(A) All patients included**							
**Study enrollment**, ***n*** **(%)**							
	Patients	14,116 (100%)	5,559 (39.38%)	8,557 (60.62%)			
	Hospitals	166	90 (54.22%)	76 (45.78%)			
	States	17					
**Stroke designation**, ***n*** **(%)**							
	Comprehensive stroke center		0 (0.00%)	1,737 (20.30%)	< 0.001^**†**^		
	Thrombectomy capable		369 (6.64%)	989 (11.56%)			
	Primary stroke center		3,678 (66.16%)	4,260 (49.78%)			
	Acute stroke ready		482 (8.67%)	280 (3.27%)			
	No designation		1,030 (18.53%)	1,291 (15.09%)			
**Baseline characteristics**							
	Gender, female, *n* (%)		2,961 (53.26%)	4,624 (54.04%)	0.3776	0.78%	NS
	Pre-mRS median [IQR]		0 [0.00, 1.00]	0.00 [0.00, 1.00]	0.2602	0	NS
	Age mean (sd)		65.49 ± 15.94	66.82 ± 16.30	< 0.001	1.33	2.03%
	NIHSS median [IQR]		2 [1.00, 6.00]	2 [0.00, 6.00]	< 0.001	0%	0%
**Time Intervals (min), median [IQR]**							
	Arrival to TeleNeuro first contact with NIR *(“Arrival-to-NIR notification”)*		89.50 [59.25, 122.00]	50.0 [40.00, 82.00]	< 0.001^**‡**^	−39.5	−44.13%
	Arrival to teleneurology stroke alert activation (“*Bias1”*)		12.56 [6.15, 26.28]	10.32 [4.75, 20.94]	< 0.001	−2.24	−17.83%
	Teleneurology stroke alert activation to first teleneurologist video login (“*Bias2”*)		3 [2.00, 5.00]	2 [1.00, 4.00]	< 0.001	−1	−0.33%
	Bias1 + Bias2		15.56	12.32		−3.24^**§**^	
**Workflow type**, ***n*** **(%)**							
	Arrival by EMS		2,840 (51.09%)	4,770 (55.74%)	< 0.001	4.65%	9.10%
	Triage/walk-in		2,719 (48.91%)	3,787 (44.26%)	< 0.001	−4.65%	−9.51%
	Hospital pre-alert by EMS, Yes		997 (17.93%)	2,134 (24.94%)	< 0.001	7.01%	39.10%
	EMS prenotification duration before arrival		5 [2.00, 9.00]	5.00 [2.00, 8.00]	0.4624	0	NS
**Thrombolytic**							
	Yes, *n* (%)		359 (6.46%)	545 (6.37%)	0.8605	−0.09%	NS
	DTN time, median [IQR]		44.00 [32.00, 57.50]	40.00 [30.00, 52.00]	0.0184	−4	−9.10%
**Advanced imaging performed (CTA or CTA/CTP)**							
	Yes, *n* (%)		2,057 (37.00%)	3,622 (42.33%)	< 0.001	5.33%	14.41%
**LVO detection rate**, ***n*** **(%)**							
	Out of total patient population, Yes		230 (4.14%)	459 (5.36%)	< 0.001	1.22%	29.47%
	Out of population that received advanced imaging		230 (11.18%)	459 (12.67%)	0.09226	1.49%	NS
**NIR rate of acceptance for MT**							
	Accepted out of total population, *n* (%)		115 (2.1%)	195 (2.3%)	< 0.0001	0.20%	9.52%
	Accepted out of population that received advanced imaging %		5.60%	5.40%	< 0.001	−0.20%	−3.57%
	% accepted out of LVO diagnosed by teleneurologist %		50%	42.27%	< 0.0001	−7.73%	−15.46%
**(B) EMS pre-alert patients excluded**							
**Study enrollment**, ***n***							
	Subjects without EMS pre-alert *(n with complete time data 99.53%)*	10,933 (77.45%)	4,539 (41.52%)	6,394 (58.48%)			
	Subjects without EMS pre-alert with suspected LVO	390	137 (3.02%)	253 (3.96%)	0.0082	0.94%	31.13%
**Time Intervals (min), median [IQR]**							
	Arrival to TeleNeuro first contact with NIR *(“Arrival-to-NIR notification”)*		95.93 [66.97, 124.77]	65.40 [45.72, 92.15]	< 0.0001	−30.53	−31.83%
	Arrival to teleneurology stroke alert activation (“*Bias1”)*		12.55 [6.16, 26.29]	10.30 [4.75, 20.95]	< 0.0001	−2.25	−17.93%
	Teleneurology stroke alert activation to first teleneurologist video login *(“Bias2”)*		3.67 [2.39, 5.45]	2.90 [1.82, 4.42]	< 0.0001	−0.77	−20.98%
	Teleneurologist first video login attempt to call to NIR to discuss possible MT for suspected LVO *(“Login-to-NIR notification”)*		74.25 [51.60, 101.22]	45.75 [32.64, 69.45]	< 0.0001^**♢**^	−28.5	−38.38%
**(C) EMS pre-alert patients only**							
**Study enrollment**, ***n*** **(%)**							
	Subjects with EMS pre-alert	3,131 (22.18%)	997 (31.84%)	2,134 (68.16%)			
	Subjects with EMS pre-alert with suspected LVO	299	93 (9.33%)	206 (9.65%)	0.8234	0.32%	NS
**Time intervals (min), median [IQR]**							
	Arrival to TeleNeuro first contact with NIR *(“Arrival-to-NIR notification”)*		104.73 [54.17, 132.70]	44.59 [39.07, 59.92]	0.0002	−60.14	−57.42%
	Teleneurologist video login to patient arrival		2.07 [1.00, 3.93]	1.89 [0.87, 3.52]	0.0297	−0.18	−8.70%
	Teleneurology stroke alert activation (SAA) to first teleneurologist video login *(“Bias2”)*		4.43 [2.98, 6.55]	4.16 [2.54, 6.28]	0.053	−0.27	NS
	Teleneurologist video login to call to NIR to discuss possible MT for suspected LVO *(“Login-to-NIR notification”)*		101.49 [51.80, 130.32]	43.19 [36.86, 59.14]	0.0011	−58.3	−57.44%

^*^NS, Not statistically significant.

^†^Grouped statistical comparison p < 0.001.

^‡^Arrival-to-NIR IQR range analysis demonstrated a smaller range for AI [non-AI IQR (59.25, 122.00) vs. AI IQR (40.00, 82.00), p = 0.0116].

^§^ The sum of Bias1 and Bias2 (−3.24 min) accounts for 8.20% of the −39.5 min reduction in the Arrival-to-NIR notification time of AI over the non-AI cohort.

^♢^Login-to-NIR IQR range analysis demonstrated a smaller range for AI [non-AI IQR (51.60, 101.22) vs. AI IQR (32.64, 69.45), p = 0.0180].

### Subgroup analysis: AI vs. non-AI by thrombectomy capability

Table 3 summarizes the results of the subgroup analyses. The predominant finding was a shorter Arrival-to-NIR notification duration in AI group regardless of whether the patient presented to a non-thrombectomy capable facility (non-TC) or a thrombectomy capable facility (TC; Table 3A). Within the non-TC subgroup, the presence of AI platform was associated with a 33 min (34.02%) median faster time in Arrival-to-NIR notification [AI: 64 min (46.00, 91.00) vs. non-AI: 97 min (62.00, 126.50), *p* < 0.001]. Within the TC subgroup the presence of AI platform was associated with a 34.0 min (43.59%) median faster time in Arrival-to-NIR notification [AI: 44.0 min (33.75, 59.00) vs. non-AI: 78 min (55.00, 95.00), *p* < 0.001]. As seen in the overall analysis, a statistically significant reduction was seen in the IQR in Arrival-to-NIR in AI vs. non-AI in both the non-TC and TC subgroups (non-TC: *p* = 0.0181, TC: *p* = 0.0148; Table 3 footnotes, line 3 and 4). A statistically faster time for Bias1 was seen for AI in both non-TC and TC subgroups; (non-TC: median delta = 1.41 min, *p* < 0.001; TC: median delta = 6.09 min, *p* < 0.001). Similarly, a statistically faster time for Bias2 was seen for AI in both non-TC and TC subgroups; (non-TC: median delta = 1 min, *p* < 0.001; TC: median delta = 1 min, *p* < 0.001). Bias1 + Bias2 accounted for 7.30% (non-TC) and 20.85% (TC) of the overall faster Arrival-to-NIR notification times in the AI cohorts (Table 3, footnotes lines 5 and 6).

**Table 3:** Subgroup analyses: effect of AI in thrombectomy capable vs. non-thrombectomy capable facilities.

		**Non-thrombectomy capable (non-TC)**					**Thrombectomy capable (TC)**				
		**Non-AI**	**AI**	***P*** **-value**	**Difference**	**% change** ^*^	**Non-AI**	**AI**	***P*** **-value**	**Difference**	**% change** ^*^
**(A) All patients included**											
**Study enrollment (total** ***n*** **=** **14,116)**		4,962 (46.38%)	5,737 (53.62%)				597 (17.47%)	2,820 (82.53%)			
**Stroke designation**, ***n*** **(%)**											
	Comprehensive stroke center	0 (0.00%)	0 (0.00%)	< 0.001^**†**^			0 (0.00%)	1,737 (61.60%)	< 0.001^**†**^		
	Thrombectomy capable	0 (0.00%)	0 (0.00%)				369 (61.81%)	989 (35.07%)			
	Primary stroke center	3,450 (69.53%)	4,166 (72.62%)				228 (38.19%)	94 (3.33%)			
	Acute stroke ready	482 (9.71%)	280 (4.88%)				0 (0.00%)	0 (0.00%)			
	No designation	1,030 (20.76%)	1,291 (22.50%)				0 (0.00%)	0 (0.00%)			
**Baseline characteristics**											
	Gender, female *n* (%)	2,667 (53.75%)	3,131 (54.58%)	0.4027	0.83%	NS	294 (49.25%)	1,493 (52.94%)	0.1101		NS
	Pre-mRS median [IQR]	0 [0.00, 1.00]	0 [0.00, 1.00]	0.117	0	NS	0.00 [0.00, 1.00]	0 [0.00, 1.00]	0.2377		NS
	Age mean yr (sd)	65.29 ± 16.00	65.41 ± 16.64	0.2578	0.12	NS	67.15 ± 15.38	69.69 ± 15.17	0.0002		
	NIHSS median [IQR]	2.00 [1.00, 5.00]	2 [0.00, 5.00]	< 0.001	0	0%	3 [1.00, 8.00]	3.00 [0.00, 9.00]	0.7289		NS
**Time Intervals (min), median [IQR]**											
	Arrival to TeleNeuro first contact with NIR *(“Arrival-to-NIR notification”)*	97 [62.00, 126.50]	64 [46.00, 91.00]	< 0.001^**‡**^	−33.0	−34.02%	78 [55.00, 95.00]	44.0 [33.75, 59.00]	< 0.001^**§**^	−34.0	−43.59%
	Arrival to teleneurology stroke alert activation *(“Bias1”)*	12.38 [5.97, 25.76]	10.97 [5.23, 21.67]	< 0.001	−1.41 min	−11.38%	14.62 [7.31, 28.80]	8.53 [3.81, 17.54]	< 0.001	−6.09	−41.66%
	Teleneurology stroke alert activation to first teleneurologist video login attempt *(“Bias2”)*	3.00 [2.00, 5.00]	2 [1.00, 4.00]	< 0.001	−1 min	−33.33%	3 [2.00, 5.00]	2.00 [1.00, 4.00]	< 0.001	−1	−33.33%
	Bias1 + Bias2 (min)				2.41^**♢**^					7.09^**#**^	
**Workflow type**, ***n*** **(%)**											
	Arrival by EMS	2,506 (50.50%)	2,811 (49.00%)	0.1249	−1.50%	NS	334 (55.95%)	1,959 (69.47%)	< 0.001	13.52%	24.16%
	Triage/walk-in	2,456 (49.50%)	2,926 (51.00%)		1.50%	NS	263 (44.05%)	861 (30.53%)		−13.52	−30.69%
	Hospital pre-alert by EMS, Yes	864 (17.41%)	966 (16.84%)	0.4467	−0.57%	NS	133 (22.28%)	1,168 (41.42%)	< 0.001	19.14	85.91%
	EMS prenotification duration before arrival	5.00 [2.00, 8.00]	4.00 [2.00, 7.00]	0.0011	−1.00	−20%	6 [3.00, 9.00]	5.00 [3.00, 9.00]	0.2233		
**Thrombolytic**											
	Yes, *n* (%)	310 (6.25%)	347 (6.05%)	0.6986	−37	NS	49 (8.21%)	198 (7.02%)	0.3524	−1.19%	NS
	DTN time, median [IQR]	44.00 [32.00, 58.00]	42.00 [32.25, 54.75]	0.6741	−2.00	NS	44.00 [33.00, 54.00]	37.50 [27.00, 48.75]	0.0125	−6.5	−14.77%
**Advanced imaging performed (CTA or CTA/CTP)**											
	Yes, *n* (%)	1,799 (36.26%)	2,182 (38.03%)	0.0604	1.77	NS	258 (43.22%)	1,440 (51.06%)	0.0006	7.84%	18.14%
**LVO detection rate**, ***n*** **(%)**											
	Out of total patient population, Yes	189 (3.81%)	260 (4.53%)	0.064	0.72%	NS	41 (6.87%)	199 (7.06%)	0.0016	0.19%	2.77%
	Out of population that received advanced imaging, Yes	189 (10.51%)	260 (11.92%)	0.1619	1.41%	NS	41 (0.71%)	199 (7.10%)	0.0001	6.39%	100.00%
**NIR rate of acceptance for MT**											
	Accepted out of total population *n* (%)	88 (1.77%)	93 (1.62%)	< 0.0001	−0.15%	−8.47%	27 (4.52%)	101 (3.58%)	0.3785	−0.94%	NS
	Accepted out of patients with advanced imaging (%)	4.89%	4.26%	< 0.0001	−0.63%	−12.87%	10.47%	7.01%	0.2000	−3.46%	NS
	Accepted out of LVO diagnosed by teleneurologist (%)	46.56%	35.77%	< 0.0001	−10.79%	−23.17%	65.85%	50.75%	0.2000	−15.10%	NS
**(B) EMS pre-alert patients excluded**											
**Study enrollment (*****n*** **=** **10,933, 77.45%)**	4,077 (82.26%)	4,754 (82.87%)				462 (77.39%)	1,640 (58.16%)			
	Pre-alert patients excluded with suspected LVO, *n* (%)	111 (2.72%)	186 (3.91%)	0.0024	1.19%	43.75%	24 (5.19%)	66 (4.02%)	0.333	−1.17%	NS
**Time Intervals (min), median [IQR]**											
	Arrival to TeleNeuro first contact with NIR *(“Arrival-to-NIR notification”)*	104.67 [74.24, 133.52]	70.15 [47.31, 94.13]	< 0.0001	−34.52	−32.98%	80.85 [60.62, 101.17]	53.77 [43.25, 86.95]	0.0144	−27.08	−33.50%
	Arrival to teleneurology stroke alert activation *(“Bias1”)*	12.38 [5.98, 25.83]	10.95 [5.23, 21.66]	< 0.0001	−1.43	−11.55%	14.77 [7.34, 28.93]	8.51 [3.85, 17.60]	< 0.0001	−6.26	−42.38%
	Teleneurology stroke alert activation to first teleneurologist video login attempt *(“Bias2”)*	3.67 [2.38, 5.45]	2.97 [1.87, 4.47]	< 0.0001	−0.7	−19.07%	3.72 [2.52, 5.47]	2.77 [1.70, 4.27]	< 0.0001	−0.95	−25.53%
	Teleneurologist video login to call to NIR to discuss possible MT for suspected LVO (“Login-to-NIR notification”)	82.11 [51.76, 110.44]	46.34 [32.76, 71.83]	< 0.0001^♡^	−35.77	−43.56%	63.45 [42.44, 76.47]	39.86 [32.26, 60.49]	0.0187^**♠**^	−23.59	−37.18%
**(C) EMS pre-alert patients only**											
**Study enrollment (*****n*** **=** **3,131, 22.18%)**	864 (47.21%)	966 (52.79%)				133 (10.22%)	1,168 (89.78%)			
	EMS pre-alert patients only with suspected LVO, *n* (%)	77 (8.91%)	74 (7.43%)	0.2791	−1.48%	NS	16 (12.03%)	132 (11.30%)	0.9151	−0.73%	NS
**Time Intervals (min), median [IQR]**											
	Arrival to TeleNeuro first contact with NIR *(“Arrival-to-NIR notification”)*	105.45 [56.53, 133.66]	50.08 [41.96, 61.85]	0.0127	−55.37	−52.51%	80.35 [51.43, 112.23]	44.00 [35.37, 59.13]	0.0817	−36.35	NS
	Teleneurologist video login to patient arrival	2.00 [1.00, 3.92]	2.00 [0.98, 3.98]	0.5614	0	NS	2.67 [1.13, 4.23]	1.71 [0.73, 3.05]	0.045	−0.96	−35.96%
	Teleneurology stroke alert activation (SAA) to first teleneurologist video login *(“Bias2”)*	4.40 [2.97, 6.52]	4.11 [2.62, 6.33]	0.1596	−0.29	−6.59%	4.75 [3.46, 6.91]	4.21 [2.50, 6.18]	0.149	−0.54	NS
	Teleneurologist video login to call to NIR to discuss possible MT for suspected LVO *(“Login-to-NIR notification”)*	103.03 [54.45, 131.43]	48.88 [39.62, 60.56]	0.0234	−54.15	−52.56%	76.87 [47.46, 109.11]	43.02 [32.86, 57.84]	0.1228	−33.85	NS

^*^NS, Not statistically significant.

^†^Grouped statistical comparison p < 0.001.

^‡^Arrival-to-NIR IQR range analysis demonstrated a significantly smaller range for non-TC AI [non-AI IQR (62.00, 126.50) vs. AI IQR (46.00, 91.00) p = 0.0181].

^§^Arrival-to-NIR IQR range analysis demonstrated a significantly smaller range for TC AI [non-AI IQR (55.00, 95.00) vs. AI IQR (33.75, 59.00), p = 0.0148].

^♢^The sum of Bias1 and Bias2 (−2.41 min) accounts for 7.30% of the −33 min reduction in the Arrival-to-NIR notification of non-TC-AI over the non-TC-non-AI cohort.

^#^The sum of Bias1 and Bias2 (−7.09 min) accounts for 20.85% of the −34 min reduction in the Arrival-to-NIR notification for the TC-AI over the TC-non-AI cohort.

^♡^Login-to-NIR IQR range analysis demonstrated a smaller range for non-TC AI [non-TC non-AI IQR (51.76, 110.44) vs. non-TC AI IQR (32.76, 71.83), p = 0.0199].

^♠^Login-to-NIR IQR range analysis demonstrated a smaller range for TC AI [TC non-AI IQR (42.44, 76.47) vs. TC AI IQR (32.26, 60.49), p = 0.0240].

There was no difference in the percentage of patients treated with IV thrombolytics between AI vs. non-AI in either the TC or non-TC subgroups. While there was no difference between AI vs. non-AI median door to needle time (DTN) in the non-TC subgroup (Table 3A), a significantly faster DTN (delta = −6.5 min) was observed in AI group within the TC subgroup [37.50 min (27.00, 48.75) vs. 44.00 min (33.00, 54.00), *p* = 0.0125].

### Effect of EMS pre-alert stroke notification

Due to 7.01% more EMS pre-alert patients in the AI group (which could theoretically lead to shorter Arrival-to-NIR notification times by having the teleneurologist on camera faster thereby expediting CTA acquisition), we next performed additional sub analyses first with all pre-alert patients excluded, then with only pre-alert patients included.

With pre-alerts excluded (Table 2B), there was a 30.53 min (31.83%) reduction in Arrival-to-NIR notification at AI facilities [AI: 65.40 min (45.72, 92.15) vs. non-AI: 95.93 min (66.97, 124.77), *p* < 0.0001]. The pre-alert excluded analysis also demonstrated only a small, albeit significant, difference for AI vs. non-AI for Bias1 and Bias2 (Table 2B). The pre-alert excluded subgroup analysis (Table 3B) showed a significant, 34.52 min (32.98%) faster Arrival-to-NIR notification in the non-TC AI subgroup [AI: 70.15 min (47.31, 94.13) vs. non-AI: 104.67 min (74.24, 133.52), *p* < 0.0001] and a 27.08 min (33.50%) faster Arrival-to-NIR notification in the TC AI subgroup [AI: 53.77 min (43.25, 86.95) vs. non-AI: 80.85 min (60.62, 101.17), *p* = 0.0144]. Importantly, in the pre-alert excluded analysis exact times from teleneurologist first video login attempt to first NIR notification could be calculated (“Login-to-NIR notification”), effectively removing Bias1 and Bias2 entirely (calculation of this metric for the all-patients-included analysis was not possible due to imbalance in the percent of pre-alerts in AI vs. non-AI with teleneurologists typically being on camera prior to patient arrival in the pre-alerted patients thus skewing the data). This analysis demonstrated a 28.5 min (38.38%) faster time in the teleneurologist Login-to-NIR notification for AI [AI: 45.75 min (32.64, 69.45) vs. non-AI: 74.25 min (51.60, 101.22), *p* < 0.0001]. Similarly, the subgroup analysis (Table 3B) demonstrated the non-TC Login-to-NIR notification time to be 35.77 min (43.56%) faster at AI [AI: 46.34 min (32.76, 71.83) vs. non-AI: 82.11 min (51.76, 110.44), *p* < 0.0001] and for TC, the Login-to-NIR notification time was 23.59 min (37.18%) faster at AI [AI: 39.86 min (32.26, 60.49) vs. non-AI: 63.45 min (42.44, 76.47), *p* = 0.0187].

Table 2C shows the results of the overall analysis with EMS pre-alert patients included only. This analysis revealed a large 60.14 min (57.42%) faster time in Arrival-to-NIR notification (*p* = 0.0002) and a 58.3 min (57.44%) faster time in Login-to-NIR notification (*p* = 0.0011) in favor of the AI cohort. In the non-TC subgroup analysis (Table 3C) highly significant faster times in the AI cohort were maintained for both time metrics, however, in the TC subgroup a significant difference was not seen in the setting of low subject number.

### Rate of LVO detection and NIR acceptance for MR

A statistically higher rate of advanced imaging was performed at AI vs. non-AI in the overall analysis (42.33 vs. 37.00%, *p* < 0.001, Table 2A). This difference was largely driven by the higher rate of advanced imaging performed in the TC subgroup (Table 3A). In the overall analysis (Table 2A), a slightly higher rate of LVO detection occurred in AI (AI: 5.36% vs. non-AI: 4.14%; *p* < 0.001). When limited to those that underwent advanced imaging (CTA or CTA/CTP), no difference in overall LVO detection was found between AI vs. non-AI. The subgroup analysis, however, did demonstrate a significantly higher rate of LVO detection among patients undergoing advanced imaging in the AI cohort within the TC subgroup (7.10 vs. 0.71%, *p* = 0.0001) but not the non-TC subgroup (NS).

Lastly, we assessed the effect of AI platform on the rate of NIR acceptance for MT. In the overall analysis (Table 2A), the presence of AI platform had an inverse effect on the rate of NIR acceptance for thrombectomy on patients with advanced imaging performed (AI: 5.4% vs. non-AI: 5.6%; *p* < 0.001) and a more robust reduction in NIR acceptance for thrombectomy in the suspected LVO population for which teleneurologists initiated contact with NIR (AI: 42.27% vs. non-AI: 50.00%; *p* < 0.0001). This critical finding was driven by a 10.79% absolute reduction (23.17% reduction) in the acceptance rate by the NIR for suspected LVO patients at non-TC (AI: 35.77% vs. non-AI: 46.56%, *p* < 0.0001, Table 3A).

## Discussion

We compared stroke workflow between a cohort of hospitals that utilized an AI-based stroke care coordination system vs. a cohort of hospitals that did not. We analyzed 14,116 stroke alerts performed over 4 months across 166 facilities in 17 states. We chose our primary outcome to be the metric of patient arrival to first contact with NIR to discuss need for thrombectomy (Arrival-to-NIR notification) as it was this measurable variable, we hypothesized, that would be most directly under the control of the AI-based care coordination platform. Ideally, measurement of CTA start time to NIR contact would have been preferable however neuro imaging start times were not available within our telemedicine database. In addition to reduced Arrival-to-NIR times, we also hypothesized that the presence of AI may lead to improved consistency of stroke care as represented by smaller values for IQR across multiple metrics. We analyzed potential imbalances in AI vs. non-AI cohorts which could skew our primary outcome measure including cohort differences in patient arrival time to teleneurology stroke alert activation (Bias1) and teleneurology stroke alert activation to first teleneurologist video login (Bias2). Lastly, we addressed the potential confounding factor of the significant 7.01% higher percentage of EMS pre-alert activations in the AI cohort (by analyzing EMS pre-alert and non-alert patients separately. This was performed to mitigate the concern that a higher percentage of EMS pre-alert patients in AI could lead to faster times to teleneurologist login, faster time to CTA acquisition, and thereby faster Arrival-to-NIR notification in AI. Significant differences did exist between the AI and non-AI cohorts: (1) 60.62% of patients overall were treated utilizing AI; (2) 100% of comprehensive centers utilized AI; (3) while an equal percentage of patients were treated with IV thrombolysis at AI and non-AI, DTN times were slightly faster at AI (4 min); and (4) a higher percentage of EMS pre-alerts occurred at AI than non-AI (58 vs. 41%).

The primary finding of this study was a 39.5 min faster median time at AI vs. non-AI for Arrival-to-NIR notification to discuss potential thrombectomy (44.13%, *p* < 0.001). In evaluating for potential biases contributing to the faster Arrival-to-NIR notification results at AI facilities, we did identify a faster arrival to stroke alert activation time (Bias1) and teleneurology stroke alert activation to teleneurologist first login attempt time (Bias2). When combined, these biases together represented 8.20% of the overall 39.50 min (44.13%) faster time in Arrival-to-NIR notification in the AI cohort. The exact magnitude of the combined biases for individual centers was not calculated but, in some cases, may have been larger given the broad IQR for Bias1 and Bias2. The relatively small median size of these biases is consistent with our conclusion that presence of AI was associated with a significantly faster time from patient Arrival-to-NIR notification at AI facilities. Importantly, the potential for a combined Bias1 and Bias2 effect significantly skewing our results was mitigated by performing a subgroup analysis in which we excluded the 22.18% of total patients that underwent EMS pre-alert (non-AI: 17.93% and AI: 24.93%). Analysis of the data with pre-alert patients removed allowed us to minimize the potential bias introduced by a higher percentage of pre-alert patients at AI facilities and permitted calculation of teleneurologist first Login-to-NIR notification thus effectively removing the Bias1 and Bias2 effects. With pre-alert patients removed, we again found a highly significant [30.53 min (31.83%), *p* < 0.0001] faster overall Arrival-to-NIR notification time in favor of AI and, importantly, a statistically significant 28.50 min (38.38%, *p* < 0.0001) faster teleneurologist Login-to-NIR notification favoring AI (Table 2B). The second major finding of this study was the fact that significantly faster Arrival-to-NIR notification time at AI facilities occurred regardless of whether the patient presented to a non-TC or TC facility [non-TC delta: −33.0 min (−34.02%) and TC delta: −34 min (43.59%), *p* < 0.001 for both]. The magnitude of the combined Bias1 and Bias2 effect in the subgroup analyses was again found to be relatively small (albeit subject to the same caveat regarding potential larger bias effect at some centers due to the wide Bias1 and Bias2 IQR values). A similar analysis wherein pre-alert patients were removed to mitigate effect of bias again revealed significantly faster Arrival-to-NIR notification for both the non-TC and TC subgroups [non-TC: 34.52 min (32.98%), *p* < 0.0001; TC: 27.08 min (33.50%), *p* = 0.0144] and a significantly reduced time in teleneurologist Login-to-NIR notification for AI at both the non-TC [35.77 min (43.56%), *p* < 0.0001] and TC subgroups [23.59 min (37.18%), *p* = 0.0187%, Table 3B].

The association between AI and consistency of stroke workflow appeared to be a particularly important finding. We found a highly significant reduction in the IQR spread at AI facilities in the global and subgroup analyses for both metrics of patient Arrival-to-NIR notification and teleneurologist Login-to-NIR notification (see Table 2 footnotes line 3 and Table 3 lines 3, 4, 7, 8). This significant reduction in the IQR spread in the AI vs. non-AI group further suggests that AI may improve uniformity of stroke care delivery. As expected, the presence of AI was not associated with an increase in the rate of thrombolysis over non-AI facilities and only a 4 min faster DTN needle time. As the decision to initiate thrombolysis is typically based on plain CT and is typically administered intravenously before CTA/CTP is performed, we would not expect the presence of AI to affect this metric. The overall 4 min faster DTN time (9.1%, *p* = 0.0184) in AI may be related to a 20.3% higher volume of comprehensive centers in the AI cohort which may operate with increased efficiency.

This study investigated potential effects of AI on critical stroke workflow metrics rather than clinical outcomes due to the multifaceted determinants of outcome and the challenges of collecting outcome data on such a large dataset. Nonetheless, the limitations of this study highlight the critical importance of developing large scale database systems connecting multi-healthcare networks in which a wide range of stroke workflow metrics, full neuroimaging time points, full patient transfer details, thrombectomy procedure metrics, and both short term and long-term outcome measures are analyzed. The development of such big data systems is essential if we are to fully vet the effects of any new technology on patient outcomes in real world settings. A significant limitation of this study is our inability to capture CTA times. This raises the potential bias in which a shorter time from patient arrival to time of CTA completion (potentially driven by the higher EMS pre-alert population in the AI cohort) may have been a driver to faster Arrival-to-NIR notification. It is however important to consider that each stroke alert at AI and non-AI facilities was executed under the watchful eye of the same groups of teleneurologist specifically charged to orchestrate stroke codes to prevent unnecessary delays in the performance of CTA. We therefore think it is unlikely that a significant delay in the performance of the CTA (thereby delaying appearance of neuroimaging on the teleneurologists' mobile app) would account for the large difference in the Arrival-to-NIR times between AI and non-AI groups. Our results did show that while the median duration of EMS prenotification prior to arrival for AI vs. non-AI was not significantly different, a significant 4.65% (*p* < 0.001) higher percentage of AI patients arrived by EMS and of all EMS patients a 7.01% (*p* < 0.001) higher percentage of AI cases experienced prehospital alert. The presence of EMS pre-hospital alert has been reported by multiple investigators to be associated with faster times for door-to-CT, door-to-needle, and door-to-mechanical thrombectomy along with improved patient outcome (Patel et al., 2011; Lin et al., 2012; Hsieh et al., 2016; Sheng et al., 2018; Fujiwara et al., 2022; Oostema et al., 2023). Therefore, we cannot exclude these imbalances in EMS metrics having some effect on our overall faster Arrival-to-NIR time for AI vs. non-AI. However, a significantly faster Arrival-to-NIR for AI vs. non-AI in the non-TC subgroup analysis (33.0 min, 34.02%, *p* < 0.001) was demonstrated despite the fact that this subgroup exhibited no difference in the percentage of patients arriving by EMS or the percent of EMS patients undergoing pre-alert. This finding would tend to argue against a large confounding effect in our results due to EMS metric imbalance. Interestingly, we also found that the largest improvement in Arrival-to NIR times correlated with those patients that exhibited both EMS pre-alert and the presence of AI (Arrival-to-NIR overall analysis improvement 57.42% (*p* = 0.0002) AI vs. non-AI; and Arrival-to-NIR improvement in non-TC subgroup 52.51% (*p* = 0.0127) AI vs. non-AI). The exact cause of this apparent interaction remains unclear. Future development of big data systems will be critical to allowing us to calculate CTA time-to-NIR-notification time, and similar metrics, that will be more recalcitrant to bias effects.

Given the extreme challenges that hospitals are facing in stroke care today, the effect that AI demonstrated in our study on the rate of patient acceptance by the NIR appears to be particularly relevant. In the overall analysis, the presence of an AI platform had an inverse effect on the rate of NIR acceptance for MT on the group of patients in which the teleneurologist contacted NIR for a suspected LVO. This negative effect of AI on NIR acceptance of LVO-identified patients appeared to be driven by patients presenting to non-TC centers [10.79% absolute reduction (−23.17%), *p* < 0.001]. While the exact reason for this phenomenon is uncertain, we hypothesize that the AI platform may impact NIR decision making by providing a unique high-quality mobile CT, CTA, and CTP review station to review outside facility neuroimaging. We hypothesize that the AI mobile platform, utilized by NIRs in this study, which allowed them to scrutinize a CTA with full MIP and 3-D rotational ability around any point in the brain may have played a significant role in improved selection of appropriate endovascular candidates. With neuroimaging confidently reviewed, the NIRs may have declined to accept for MT what would otherwise have been a “futile transfer” upon patient arrival at the TC center. The reduction in futile transfers has never been more important than now for both hub and spoke hospitals (Mullen et al.). With hub hospitals across the United States experiencing critical staffing shortages, the ability to limit transfers to those patients most in need of higher level of care is of paramount importance. Similarly, the need to shut down futile transfers, thereby driving spoke hospital revenue, is now critically important for countless referral hospitals teetering on financial collapse (American Hospital Association, 2022; Davenport, 2023; Thompson, 2023; Mullen et al.). The positive results of the “large core” interventional trials enrolling patients with ASPECTS scores 3–5, will likely lead to a change in guidelines such that more patients with large infarcts are transferred and undergo thrombectomy (Yoshimura et al., 2022; Huo et al., 2023; Sarraj et al., 2023). Despite this expected paradigm shift, the goal of getting every patient admitted expediently to a center able to maximize their outcome will not change. Therefore, AI will likely remain a critical tool in our armamentarium for LVO detection, provider communication, and treatment facilitation.

Our results beg the question: are these findings specific to the practice of telemedicine or are they more widely generalizable? Stroke workflow at telemedicine-based facilities is directly mirrored after highly efficient in-person neurologist-based facilities with only minor variations. As such, when assessing the potential effect of AI on stroke workflow, our findings are less likely to represent a “telemedicine-specific” effect. The presence of AI platform did not increase the LVO detection rate across the total population of patients that underwent advanced imaging (Table 2A). A small, but significant, increase was seen in the rate of LVO detection among those patients undergoing advanced imaging in the TC AI vs. non-AI subgroups (7.10 vs. 0.715%, *p* = 0.0001) but not the non-TC subgroup (Table 3A). The lack of difference in the overall LVO detection rate in our study between AI and non-AI may have been attributable to both a treating neurologist and a radiologist reviewing all neuroimaging emergently. At other hospital systems without this level of intense emergent neuroimaging scrutiny a greater LVO detection rate with AI may be more likely. Previous investigators have reported higher levels of LVO detection rates commensurate with the level of neuro-specific training of their reviewing physicians (Karamchandani et al., 2022).

## Conclusion

In conclusion, despite our modern-day deeper understanding of “time is brain” (now incorporating the concept of “slow progressors and fast progressors”), we still cannot predict the specific infarct rate for any individual LVO patient. As such, all potential MT patients should be treated as fast progressors with laser focus by the ED stroke team on rapid LVO detection and getting appropriate neuroimaging into the hands of the treating NIR physicians. Numerous studies have been published giving us great insight into the highly time dependent nature of MT eligibility and outcomes (Sun et al., 2013; Goyal et al., 2015, 2016; Jovin et al., 2015; Saver et al., 2015; Albers et al., 2018; Mulder et al., 2018; Nogueira et al., 2018; Carrion-Penagos et al., 2022). While it was not the purpose of this study to prove “time is brain,” the degree of potential time savings reported in this study would be considered by most clinicians to be in the highly relevant range. In fact, delays in contacting the neurointerventionalist have been estimated to comprise ~57% of the entire patient door in/door out time at spoke hospitals (Sun et al., 2013). The results of this large multicenter study, when combined with the results of previous studies, support the adoption of advanced AI-based care coordination platforms with automated LVO detection, neuroimaging review, and communication systems into the armamentarium of acute stroke care hospitals. Most importantly, this study underscores the critical need to develop “big data” systems linking multi-hospital networks to capture detailed data on stroke workflow, neuro imaging times, specifics of treatments, and patient outcomes. Only in this way will we be able to fully vet the potential benefit of AI (and other emerging technologies), expedite adoption, and maximize the outcomes of our patients.

## Data availability statement

The original contributions presented in the study are included in the article/supplementary material, further inquiries can be directed to the corresponding author.

## Ethics statement

The studies involving humans were approved by TeleSpecialists IRB, Fort Myers, FL, USA. The studies were conducted in accordance with the local legislation and institutional requirements. The Ethics Committee/Institutional Review Board waived the requirement of written informed consent for participation from the participants or the participants' legal guardians/next of kin because, this was purely a retrospective analysis of data acquired from the TeleSpecialist's database.

## Author contributions

TD: Conceptualization, Funding acquisition, Methodology, Writing – original draft, Writing – review & editing. LG: Conceptualization, Data curation, Formal analysis, Methodology, Project administration, Supervision, Visualization, Writing – original draft, Writing – review & editing. OC: Data curation, Formal analysis, Visualization, Writing – review & editing. GH: Data curation, Formal analysis, Supervision, Writing – review & editing, Conceptualization, Investigation, Methodology, Validation, Visualization. MF: Conceptualization, Data curation, Investigation, Supervision, Writing – review & editing, Resources. AA: Data curation, Writing – review & editing, Supervision, Conceptualization, Formal analysis, Methodology, Project administration, Validation. CB: Data curation, Supervision, Writing – review & editing, Resources. HA: Data curation, Supervision, Writing – review & editing, Formal analysis. TS: Conceptualization, Data curation, Formal analysis, Funding acquisition, Investigation, Methodology, Resources, Supervision, Validation, Visualization, Writing – original draft, Writing – review & editing.

## References

[B1] Albers G. Marks M. Kemp S. Christensen S. Tsai J. P. Ortega-Gutierrez S.. (2018). Thrombectomy for stroke at 6 to 16 hours with selection by perfusion imaging. N. Engl. J. Med. 378, 708–718. 10.1056/NEJMoa1713973 29364767 PMC6590673

[B2] American Hospital Association (2022). AHA Report: Rural Hospital Closures Threaten Access. Solutions to Preserve Care in Local Communities. American Hospital Association. Available online at: https://www.aha.org/system/files/media/file/2022/09/rural-hospital-closures-threaten-access-report.pdf (accessed June 1, 2023).

[B3] Aroor S. R. Asif K. S. Potter-Vig J. Sharma A. Menon B. K. Inoa V.. (2022). Mechanical thrombectomy access for all? Challenges in increasing endovascular treatment for acute ischemic stroke in the United States. J. Stroke 24, 41–48. 10.5853/jos.2021.03909 35135058 PMC8829477

[B4] Carrion-Penagos J. Thind S. Coleman E. Brorson J. McKoy C. Mendelson S.. (2022). Endovascular therapy delay for acute large vessel occlusion (LVO) is associated with worse functional outcome and increased mortality. AAN Abstract 2022:13.004. 10.1212/WNL.98.18_supplement.30931452015

[B5] Danziger R. Tan C. Churilov L. Mitchell P. Dowling R. Bush S.. (2021). Intrinsic hospital factors: overlooked cause for variations in delay to transfer for endovascular thrombectomy. J. NeuroIntervent. Surg. 13, 968–973. 10.1136/neurintsurg-2020-016836 33593802

[B6] Davenport K. (2023). New Medicare Designation Could Prevent Closure of Struggling Rural Hospitals. National Conference of State Legislatures. Available online at: https://www.ncsl.org/state-legislatures-news/details/new-medicare-designation-could-prevent-closure-of-struggling-rural-hospitals (accessed June 1, 2023).

[B7] Devlin T. Shah R. Patterson J. Owen J. Paradis O. Heath G. (2020). Distinction: Utilization of Applied Artificial Intelligence to Facilitate LVO Detection and Synchronizing Workflow to Improve Time to Treatment in High-Volume Hub & Stroke Networks. World Stroke Organization.

[B8] Elijovich L. Iii D. D. Nickele C. Alexandrov A. Inoa-Acosta V. Arthur A. S.. (2022). Automated emergent large vessel occlusion detection by artificial intelligence improves stroke workflow in a hub and spoke stroke system of care. J. Neurointerv. Surg. 14, 704–708. 10.1136/neurintsurg-2021-017714 34417344

[B9] Figurelle M. Meyer D. Perrinez E. Santiago-Dieppa D. R. Khalessi A. A. Bolar D. S.. (2023). Viz.ai implementation of stroke augmented intelligence and communications platform to improve indicators and outcomes for a comprehensive stroke center and network. Am. J. Neuroradiol. 44, 47–53. 10.3174/ajnr.A7716 36574318 PMC9835916

[B10] Fujiwara S. Kuroda T. Matsuoka Y. Ohara N. Imamura H. Yamamoto Y.. (2022). Prehospital stroke notification and endovascular therapy for large vessel occlusion: a retrospective cohort study. Sci. Rep. 12:10107. 10.1038/s41598-022-14399-0 35710934 PMC9203518

[B11] Goyal M. Demchuk A. Bijoy K. Eesa M. Rempel J. L. Thornton J.. (2015). Randomized assessment of rapid endovascular treatment of ischemic stroke. N. Engl. J. Med. 372, 1019–1030. 10.1056/NEJMoa1414905 25671798

[B12] Goyal M. Menon B. K. van Zwam W. H. Dippel D. W. J. Mitchell P. J. Demchuk A. M.. (2016). Endovascular thrombectomy after large-vessel ischaemic stroke: a meta-analysis of individual patient data from five randomized trials. Lancet 387, 1723–1731. 10.1016/S0140-6736(16)00163-X 26898852

[B13] Hassan A. Ringheanu V. Preston L. Tekle W. G. (2022b). Artificial intelligence-parallel stroke workflow tool improves reperfusion rates and door-in to puncture interval. Stroke Vasc. Interv. Neurol. 2:e000224. 10.1161/SVIN.121.000224 PMC12778824 41584017

[B14] Hassan A. Ringheanu V. Tekle W. (2022a). The implementation of artificial intelligence significantly reduces door-in-door-out times in a primary care center prior to transfer. Interv. Neuroradiol. 2022:15910199221122848. 10.1177/15910199221122848 36017543 PMC10680953

[B15] Hsieh M.-J. Tang S. C. Chiang W. C. Tsai L. K. Jeng J. S. Ma M. H. M.. (2016). Effect of prehospital notification on acute stroke care: a multicenter study. SJTREM 24:57. 10.1186/s13049-016-0251-2 27121501 PMC4847216

[B16] Huo X. Ma G. Tong X. Zhang X. Pan Y. Nguyen T. N.. (2023). Trial of endovascular therapy for acute ischemic stroke with large infarct. N. Engl. J. Med. 388, 1272–1283. 10.1056/NEJMoa2213379 36762852

[B17] Jovin T. Chamorro A. Cobo E. de Miquel M. A. Molina C. A. Rovira A.. (2015). Thrombectomy within 8 hours after symptom onset in ischemic stroke. N. Engl. J. Med. 372, 2296–2306. 10.1056/NEJMoa1503780 25882510

[B18] Karamchandani R. Helms A. Satyanarayana S. Yang H. Clemente J. D. Defilipp G.. (2022). Automated detection of intracranial large vessel occlusions using Viz.ai software: experience in a large, integrated stroke network. Brain Behav. 13:e2808. 10.1002/brb3.2808 36457286 PMC9847593

[B19] Lachkhem L. Rican S. Minvielle E. (2018). Understanding delays in acute stroke care: a systematic review of reviews. Eur. J. Publ. Health 28, 426–433. 10.1093/eurpub/cky066 29790991

[B20] Lin C. Peterson E. Smith E. Saver J. L. Liang L. Xian Y.. (2012). Emergency medical service hospital prenotification is associated with improved evaluation and treatment of acute ischemic stroke. Circ. Cardiovasc. Qual. Outcomes 5, 514–522. 10.1161/CIRCOUTCOMES.112.965210 22787065

[B21] Lotan E. (2021). Emerging Artificial Intelligence Imaging Applications for Stroke Interventions. Am. J. Neuroradiol. 42, 255–256. 10.3174/ajnr.A6902 33384290 PMC7872176

[B22] McTaggart R. A. Yaghi S. Cutting S. M. Hemendinger M. Baird G. L. Haas R. A.. (2017). Association of a primary stroke center protocol for suspected stroke by large-vessel occlusion with efficiency of care and patient outcomes. J. Am. Med. Assoc. Neurol. 74, 793–800. 10.1001/jamaneurol.2017.0477 28492918 PMC5710532

[B23] Morey J. R. Zhang X. Yaeger K. A. Fiano E. Marayati N. F. Kellner C. P.. (2021). Real-world experience with artificial intelligence-based triage in transferred large vessel occlusion stroke patients. Cerebrovasc. Dis. 50, 450–455. 10.1159/000515320 33849032

[B24] Mulder M. Jansen I. Goldhoornb R. (2018). Time to endovascular treatment and outcome in acute ischemic stroke MR CLEAN registry results. Circulation 138, 232–240. 10.1161/CIRCULATIONAHA.117.032600 29581124

[B25] Mullen D. Avila A. Mowzoon N. Optimization of acute stroke-related hospital finances utilizing artificial intelligence. J. Healthc. Manag. .

[B26] Murray N. Unberath M. Hager G. Hui F. K. (2020). Artificial intelligence to diagnose ischemic stroke and identify large vessel occlusions: a systematic review. J. NeuroIntervent. Surg. 12, 156–164. 10.1136/neurintsurg-2019-015135 31594798

[B27] Nogueira R. Jadhav A. Haussen D. Bonafe A. Budzik R. F. Bhuva P.. (2018). Thrombectomy 6 to 24 hours after stroke with a mismatch between deficit and infarct. N. Engl. J. Med. 378, 11–21. 10.1056/NEJMoa1706442 29129157

[B28] Oostema J. A. Nickles A. Allen J. Ibrahim G. Luo Z. Reeves M. J. (2023). Emergency medical services compliance with prehospital stroke quality metrics is associated with faster stroke evaluation and treatment. Stroke 55, 101–109. 10.1161/STROKEAHA.123.043846 38134248

[B29] Patel M. Rose K. O'Brien E. Rosamond W. D. (2011). Prehospital notification by emergency medical services reduces delays in stroke evaluation. Stroke 42, 2263–2268. 10.1161/STROKEAHA.110.605857 21659638 PMC3970287

[B30] Sarraj A. Hassan A. Abraham M. Ortega-Gutierrez S. Kasner S. E. Hussain M. S.. (2023). Trial of endovascular thrombectomy for large ischmic stroke. N. Engl. J. Med. 388, 1259–1271. 10.1056/NEJMoa2214403 36762865

[B31] Saver J. L. Goyal M. Bonafe A. Diener H. C. Levy E. I. Pereira V. M.. (2015). Stent-retriever thrombectomy after intravenous t-PA vs. t-PA alone in stroke. N. Engl. J. Med. 372, 2285–2295. 10.1056/NEJMoa1415061 25882376

[B32] Sheng Z. Zhang J. Shang M. Zhong G. Chen Z. Lin L.. (2018). Prehospital notification procedure improves stroke outcome by shortening onset to needle time in Chinese urban area. Aging Dis. 3, 426–434. 10.14336/AD.2017.0601 29896430 PMC5988597

[B33] Sun C. H. J. Nogueira R. G. Glenn B. A. Connelly K. Zimmermann S. Anda K.. (2013). “Picture to puncture” a novel time metric to enhance outcomes in patients transferred for endovascular reperfusion in acute ischemic stroke. Stroke 127, 1139–1148. 10.1161/CIRCULATIONAHA.112.000506 23393011

[B34] Thompson D. (2023). Hundreds of Hospitals Could Close Across Rural America. US News and World Report. Available online at: https://www.usnews.com/news/health-news/articles/2023-01-16/hundreds-of-hospitals-could-close-across-rural-america (accessed June 1, 2023).

[B35] Yoshimura S. Sakai N. Yamagami H. Uchida K. Beppu M. Toyoda K.. (2022). Endovascular therapy for acute stroke with a large ischemic region. N. Engl. J. Med. 386, 1303–1313. 10.1056/NEJMoa2118191 35138767

